# The importance of self-efficacy and negative affect for neurofeedback success for central neuropathic pain after a spinal cord injury

**DOI:** 10.1038/s41598-022-15213-7

**Published:** 2022-06-29

**Authors:** Krithika Anil, Sara Demain, Jane Burridge, David Simpson, Julian Taylor, Imogen Cotter, Aleksandra Vuckovic

**Affiliations:** 1 Faculty of Engineering and Physical Sciences, University of Southampton, Southampton, UK; 2 Faculty of Health and Human Sciences, University of Plymouth, Plymouth, UK; 3 School of Health Sciences, University of Southampton, Southampton, UK; 4 Sensorimotor Function Group, Hospital Nacional de Parapléjicos, SESCAM, Toledo, Spain; 5 Harris Manchester College, University of Oxford, Oxford, UK; 6 Department of Clinical Psychology, National Spinal Injuries Centre, Stoke Mandeville Hospital, Aylesbury, UK; 7 Department of Biomedical Engineering, School of Engineering, University of Glasgow, Glasgow, UK

**Keywords:** Health care, Neurology, Cognitive neuroscience, Learning and memory

## Abstract

EEG-based neurofeedback uses mental behaviours (MB) to enable voluntary self-modulation of brain activity, and has potential to relieve central neuropathic pain (CNP) after a spinal cord injury (SCI). This study aimed to understand neurofeedback learning and the relationship between MB and neurofeedback success. Twenty-five non-CNP participants and ten CNP participants received neurofeedback training (reinforcing 9–12 Hz; suppressing 4–8 Hz and 20–30 Hz) on four visits. Participants were interviewed about the MB they used after each visit. Questionnaires examined the following factors: self-efficacy, locus of control, motivation, and workload of neurofeedback. MB were grouped into mental strategies (a goal-directed mental action) and affect (emotional experience during neurofeedback). Successful non-CNP participants significantly used more imagination-related MS and reported more negative affect compared to successful CNP participants. However, no mental strategy was clearly associated with neurofeedback success. There was some association between the lack of success and negative affect. Self-efficacy was moderately correlated with neurofeedback success (r = < 0.587, p = < 0.020), whereas locus of control, motivation, and workload had low, non-significant correlations (r < 0.300, p > 0.05). Affect may be more important than mental strategies for a successful neurofeedback performance. Self-efficacy was associated with neurofeedback success, suggesting that increasing confidence in one’s neurofeedback abilities may improve neurofeedback performance.

## Introduction

Neurofeedback training is a neuromodulation method where an individual attempts to voluntarily regulate their own brain activity to induce a clinical outcome, such as pain relief^1^ or anxiety reduction^2^. Neurofeedback based on upregulation of alpha (8–12 Hz) of electroencephalography (EEG) signal, has been tested in numerous studies in both healthy people and patient population. A recent double blinded, randomised study based on alpha neurofeedback upregulation from electrode locations P3 and P4, over a period of 12 sessions, showed that only active group selectively increased the alpha band power and successful participants reported increased relaxation and feeling of control and reduced anxiety^3^. It is believed that abnormal alpha band power is a marker of chronic pain of neuropathic origin^4^, or even of any chronic pain^5^, making a neurofeedback particularly appealing for treatment of chronic pain. Theta band (4–8 Hz) is also affected in people with neuropathic pain of central origin, and that is attributed to phenomena called thalamocortical dysrhythmia^6^. Thalamocortical dysrhythmia is caused by the hyperpolarisation of thalamic neurons and low threshold calcium spike firing. This exerts a rhythmic influence on thalamo-cortical modules in the theta frequency band, keeping these structures in the state of reduced activity. Thus, patients suffering from central neuropathic pain have increased theta band power^6,7^. In patient treated for central neuropathic pain with thalamocortical dysrhythmia, alpha power reduced in those patients in which pain was reduced^7^.

Past research examining EEG for pain relief has aimed to supress theta and higher beta (20–30 Hz) and reinforce alpha or lower beta power. For example, Jensen et al.^1^ reinforced alpha (8–12 Hz) that resulted in pain reduction. Similarly, Kayiran et al.^8^ supressed theta (3–8 Hz) while reinforcing low beta (13–15 Hz) that also resulted in pain reduction. A recent meta-analysis found that neurofeedback based on alpha band upregulation result in clinically meaningful reduction of pain but concluded that more studies are required to confirm the results^9^.

TCD has also been observed in resting brain states of those with central neuropathic pain (CNP) after a spinal cord injury (SCI)^7^. Our team’s previous research has shown that those with CNP after SCI had a greater event related desynchronization during motor imagery compared to those with no CNP after SCI and healthy individuals^10,11^. Early stage research found that this event related desynchronization could be reversed using neurofeedback and had the potential to produce pain relief^12–14^. This resulted in the development of a neurofeedback protocol to reduce CNP after SCI, and early evidence showed that long-term neurofeedback training led to a 30% reduction in pain scores^13–15^. However, neurofeedback studies typically find that some users are not able to control their brain activity^16^ and the reasons are unclear. A systematic review of 11 studies^17^ found that between 16–57% of participants were unsuccessful in regulating their brain activity. Understanding what mental behaviours (MB) are used by successful neurofeedback participants may increase the success rate in controlling brain activity and hence pain reduction. The present paper aims to contribute to this development.

Kadosh and Staunton^16^ systematically reviewed sixteen studies examining behavioural factors as a predictor of neurofeedback performance. They concluded that behavioural factors play a crucial role in neurofeedback learning but the use of heterogenous measures of MB generated ambiguity. For example, Nijboer et al.^18^ found that a general positive mood predicted neurofeedback success while Diaz et al.^19^ found no relationship between anxiety and success. A mixed-methods approach using quantitative and qualitative methods may elucidate this behavioural complexity.

Kober et al.^20^ and Nan et al.^21^ used a qualitative approach by categorising mental strategies derived from participant-written notes of their MB. Kober et al.^20^ concluded that having no strategy (i.e. strategies that could not be verbalised or described) was more effective than having a strategy (i.e. strategies that could be verbalised or described) when regulating sensory-motor rhythm activity (13–15 Hz over the motor cortex). However, Nan et al.^21^ concluded that strategies related to positive thinking were more effective when regulating alpha activity. These studies suggest that different neurofeedback protocols may require different MB. While participant-written notes provide valuable information, one-to-one interviews may allow further probing and produce more in-depth MB data.

Combining qualitative interviews with quantitative assessment of behavioural factors associated with learning, such as locus of control (LoC) and self-efficacy (SE), may further address the inconsistencies in the neurofeedback literature^22–25^. LoC is the extent to which individuals believe they have control over situations and events. SE is an individual’s belief in their own ability to succeed. Neurofeedback requires active user participation, suggesting that LoC and SE may influence neurofeedback learning and performance. Motivation and perceived difficulty of a task are also factors associated with general learning^26–29^. Motivation is an important driver behind behaviour required to accomplish a task^30–32^ and is thus likely to also be important in neurofeedback. Neurofeedback learning is considered a difficult task to achieve^17,33^, which may influence engagement in neurofeedback training.

This study is part of a larger research programme to develop a neurofeedback protocol to relieve CNP after SCI^13–15^. The current study aims to understand which MB lead to neurofeedback success with this protocol by answering the following research questions: (1) “what MB, including affect (emotional experience during neurofeedback), are associated with success at neurofeedback?” and (2) “what are the relationships between general learning factors (i.e. LoC, SE, motivation, and difficulty) and neurofeedback performance?” The current study further aimed to identify differences in MBs between healthy controls and patients with CNP. We wished to assess if MB used by individuals with CNP after SCI were specific to their health condition, where their pain is likely a persistent and constant presence. It may be that individuals with CNP after SCI were primed to develop MB related to their pain condition, which may be due to the large impact of pain on their lives^34^, the knowledge that the neurofeedback targets their pain, or for some other reason. If we can identify the successful MB used by people with CNP after SCI, we may be able to tailor future neurofeedback guidance for this population. Additionally, the context of pain for the CNP participants may have impacted on neurofeedback success rates. Therefore, differences between these patient groups and healthy controls would be important in directing future research on neurofeedback learning, where the sampling of just one group (e.g. just healthy controls) can skew results^35,36^.

## Method

### Participants

Table 1 shows the recruitment criteria for non-CNP participants and CNP participants. Non-CNP participants were recruited face-to-face and through poster advertisements from a UK university (students and staff). CNP participants were recruited from the spinal unit of a UK hospital via the health care staff and poster advertisements. Potential CNP participants were included based on their clinical report and a score of at least 4 on the Douleur Neuropathique 4 (DN4)^37^, a screening tool used to identify the likelihood that an individual is experiencing neuropathic pain.

**Table 1:** Participant inclusion/exclusion criteria.

Inclusion criteria	Exclusion criteria
**Non-CNP participants**	
Must be at least 18 years old	Current or history of any chronic pain conditions
	Current or history of brain injury
	Current or history of a neurological condition
**Participants with CNP**	
Must be at least 18 years old	Current or history of brain injury
Must be at least 1-year post spinal cord injury	Current or history of a neurological condition other than CNP after SCI
Must have at least 6 months of treatment history for CNP	
Must report pain intensity greater than or equal to five on a numerical rating scale (0—no pain, 10—worst pain)	

*CNP* central neuropathic pain, *SCI* spinal cord injury.

### EEG recording and neurofeedback protocol

**Figure 1:**
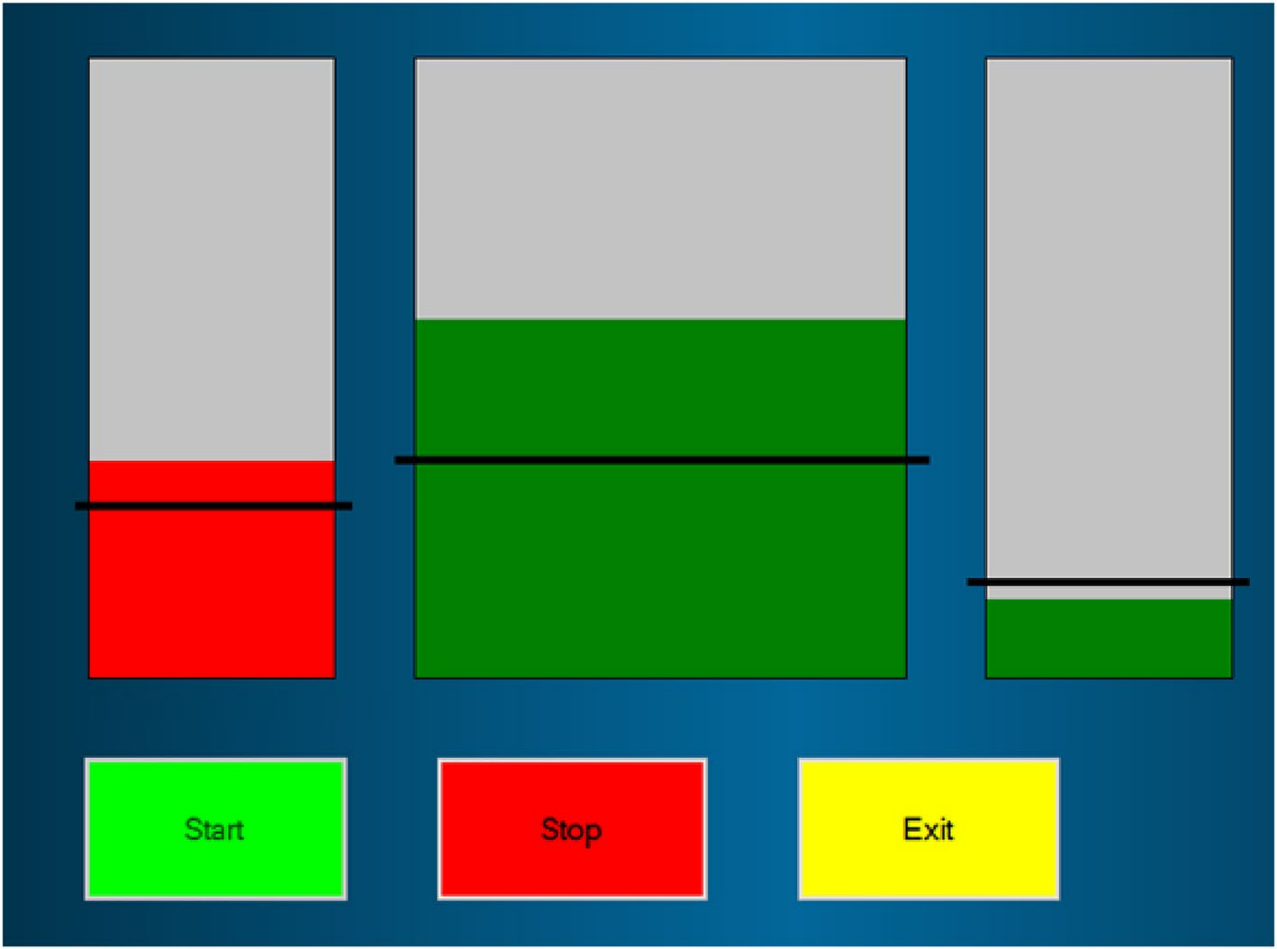
Visual interface showing three bars indicating users’ EEG activity, from left to right: theta, alpha, and higher beta; black lines indicate threshold for illustrative purposes only.

EEG was recorded using the Emotiv EPOC Model 1.0 headset using a single channel at C4. Sampling frequency was 128 Hz and impedance was set under 10 kΩ. Two reference electrodes were placed in the parietal region, above the ears for CMS/DRL noise cancellation. EEG feedback was given to participants from C4, above the primary-motor cortex. C4 was chosen after examination and comparison with three other protocols in previous research, which showed that C4 was linked with the greatest pain relief as reported by CNP participants^10,13^. The EEG signal was filtered in real time in theta (1–4 Hz), alpha (9–12 Hz), higher beta (20–30 Hz) and broadband (1–30 Hz) using 5th order Butterworth filter. EEG power was calculated over 0.5 s long windows updated after each sample. Relative power was calculated as a power in the selected frequency band over the broadband power and was expressed in percentage 0–100%. A computer tablet with Windows 10, connected to the Emotiv headset via Bluetooth, was used to visually display EEG activity to participants in the form of three bars (see Fig. 1): each bar represented the relative EEG power in specific frequency band: theta, alpha, and higher beta from left to right. For more details about signal analysis see Vuckovic et al.^14^. The middle bar (alpha) was wider than the other bars as participants in previous research reported that this bar was easier to control than other bars^13^. The neurofeedback protocol encouraged users to reinforce alpha, and supress theta and higher beta. The training threshold was set to 110% of the average power of their baseline alpha, and to 90% of the average power of their baseline theta and higher beta. When participants were successful (respectively increasing or decreasing the average power by at least 10%) the bars turned greed indicating correct control.

**Figure 2:**
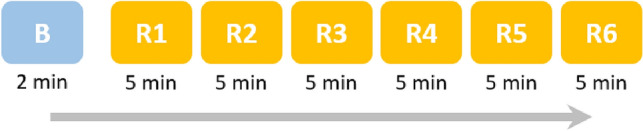
Flow diagram of neurofeedback training in a single visit. B = Baseline before the neurofeedback training. R1… R6 = First neurofeedback training run… sixth neurofeedback training run.

Participants were asked to attend the study site for four visits at least once per week; during which they completed six 5-min neurofeedback training sessions (runs; see Fig. 2). Resting EEG baseline with eyes open was measured for 2 min before neurofeedback training began. Participants were instructed to ‘turn the bars green using whatever mental strategy they prefer’. Each run involved the participant attempting to control the three bars correctly for 5 min using trial-and-error.

### Procedure

The following procedure describes each of the four visits. Participants were asked to complete the following questionnaires: the General SE scale^38^, the Health LoC scale (for CNP participants only)^39^, the General LoC scale (for non-CNP participants only)^40^, the NASA Task Load Index^41^, and a numerical rating scale for motivation (scale of 0 to 10, where 0 indicates the lowest point of motivation and 10 indicates the highest point of motivation). The General SE scale produced a single score ranging from 10 to 40, a higher score indicated higher SE. The Health LoC scale consists of four dimensions: internal LoC, perception of powerful others, perception of doctors, and perception of fate. The internal and fate dimensions have scores ranging from 6 to 36, and the powerful others and doctors dimensions have scores ranging from 3 to 18. Higher scores indicate higher levels of perceived control in that dimension. The General LoC scale produced the same dimensions as the Health LoC excluding perceptions of doctors, where all scores range from 0 to 48. The NASA Task Load Index produced a single score ranging from 0 to 100, a higher score indicates a higher task load (i.e. increased task difficulty). Participants completed all questionnaires except the NASA Task Load Index before the neurofeedback training. The NASA Task Load Index was completed after the neurofeedback training.

Participants were asked to sit comfortably in a chair while the researcher applied the Emotiv headset and explained the neurofeedback procedure. They were asked to sit as still as possible and keep looking at a cross on the screen in front of them while baseline measurements were recorded for 2 min. Participants were then informed what they would see on screen (i.e. the bars) during the neurofeedback training and were given instructions to sit as still as possible while using whatever mental strategy they preferred to try and make the bars green. No other guidance or instructions were given in order to reduce the risk of researcher bias and assumptions on neurofeedback learning. Participants were informed that they could take a break between each run. After the neurofeedback training, the Emotiv headset was removed and the participant was asked to complete the NASA Task Load Index^41^. Participants were then interviewed about their MB during neurofeedback training using a semi-structured interview schedule (see supplementary material “SM1—Interview Analysis Information”).

### Off-line EEG and neurofeedback performance analysis

EEG data were visually inspected and sections containing artefacts removed; EEG data with more than 30% of data removed were excluded from analysis. The averaged power of each frequency band at baseline and during training were compared to calculate percentage change in EEG power of each frequency band^13^. To determine change in power, the following steps were taken: (1) the absolute power of the relevant frequency bands was first calculated using Eq. (1), which calculates the average power of the signal in a specified frequency band. (2) Relative power (the absolute power of the relevant frequency band in relation to the absolute power of all the frequency bands in that EEG recording) was calculated using Eq. (2); this was done for both baseline EEG and EEG during neurofeedback training. (3) Change in power was then calculated using Eq. (3).

Equation (1) Average power of a signal for a specified frequency band

1 $$P_{\epsilon } (f) = \frac{{\mathop \sum \nolimits_{i = 1}^{n} x_{f}^{2} (i)}}{n}$$

where: *x*_*f*_: the filtered EEG signal in the frequency band f, n: The length of the signal (*x*) in samples, *i* = 1…n.

Equation (2) Relative power of a frequency band in relation to power of all frequency bands

2 $$P_{r} (f) = \frac{{P_{\epsilon } (f)}}{{\mathop \sum \nolimits_{f = f1}^{f2} P_{\epsilon } (f)}}$$

where: ${ }P_{\epsilon } (f)$: Absolute power of specific frequency (from Eq. 1), $P_{\epsilon } (f)$: Absolute power of specific frequency band; f1 and f2 are lower and higher frequencies of the selected frequency band respectively (1–35 Hz; a upper threshold of 35 Hz instead of 30 Hz was chosen to account for any individual differences in higher beta that may slightly exceed 30 Hz for offline analysis).

Equation (3) Relative change in power

3 $$P_{c} = \left( {\left( {\frac{{P_{r,t} }}{{P_{r,b} }}} \right) - 1} \right) \times 100$$

where: *P*_*r,t*_: relative power of the signal during neurofeedback training, *P*_*r,b*_: relative power of the signal during baseline, *P*_*c*_: change in power.

Participants were identified as ‘successful’ if they met all the following criteria: (1) Percentage change in PSD across the targeted frequency band increased for alpha and/or decreased for theta and/or beta), (2) participants reported actively participating in the neurofeedback task and (3) were able to correctly perceive whether or not they were successful. Some participants focussed on only one bar; in this case ‘success’ related to relevant average change in power in that specific bar. The second criterion was needed as some participants passively observed the bars with no intention of control. The reason for including participant perception as part of the success criteria is to ensure that participants are not only controlling their EEG activity correctly, but that they are knowingly and actively inducing this control. All other participants were categorised as “unsuccessful”.

### Interview analysis

Interview data were analysed using thematic analysis and a framework model. Thematic analysis^42^ is a qualitative method that identifies patterned meaning across a dataset, using codes to identify common “themes”. Framework analysis^42^ is a qualitative method that aids in organising and summarising existing data codes, where these codes are applied across interviews to identify if the same codes can be found in these interviews. Themes were constructed based on the relationship of the codes to EEG activity; for example, motor imagery is seen as a distinct mental activity that specifically changes EEG activity. Please see supplementary material “SM1—Interview Analysis Information” for details of this qualitative analysis, including development of the themes.

### Statistical analysis

Statistical analyses were only conducted for questionnaire data, where questionnaire scores (i.e. SE, LoC, motivation, and workload) were correlated with participants’ success status (successful/unsuccessful) using Pearson’s point–biserial correlation (normal distribution confirmed using the Shapiro–Wilk test and Q–Q plots). LoC correlation was only conducted for non-CNP participants because CNP participants completed a different condition-specific LoC questionnaire, and the sample size (n = 10) was too small. To understand if LoC had a relationship with neurofeedback success in CNP participants, the trend in the different LoC patterns were compared via visual inspection. Descriptive statistical analysis was carried out. Due to the complexity of the data (e.g. multiple MB used by participants) and potentially misleading results from differences in the number of participants using each MB (identified by the qualitative analysis), our statisticians’ advice was that inferential statistical analysis was not appropriate in the current exploratory study. Follow on work building on the current results and a larger sample should include such inferential analysis.

### Ethical approval

The Faculty of Engineering and the Environment, University of Southampton, UK (reference: 30254), and the NHS Research Ethics Committee, UK (reference: 18/SC/0244) provided ethical approval for this study; this ethical process is in accordance with the Declaration of Helsinki. Written, informed consent was obtained for all participants before the study began. Participants were given monetary incentives (£10/visit for non-CNP participants, and travel cover up to £50/visit for participants with CNP) to volunteer for the study. Participants with CNP could speak to a clinical psychologist at the UK hospital if they were distressed in any way by participation.

### Consent to publication

As part of the written and informed consent, all participants were informed that all results from their participation would be anonymously published.

## Results

### Participant traits and success rates

**Figure 3:**
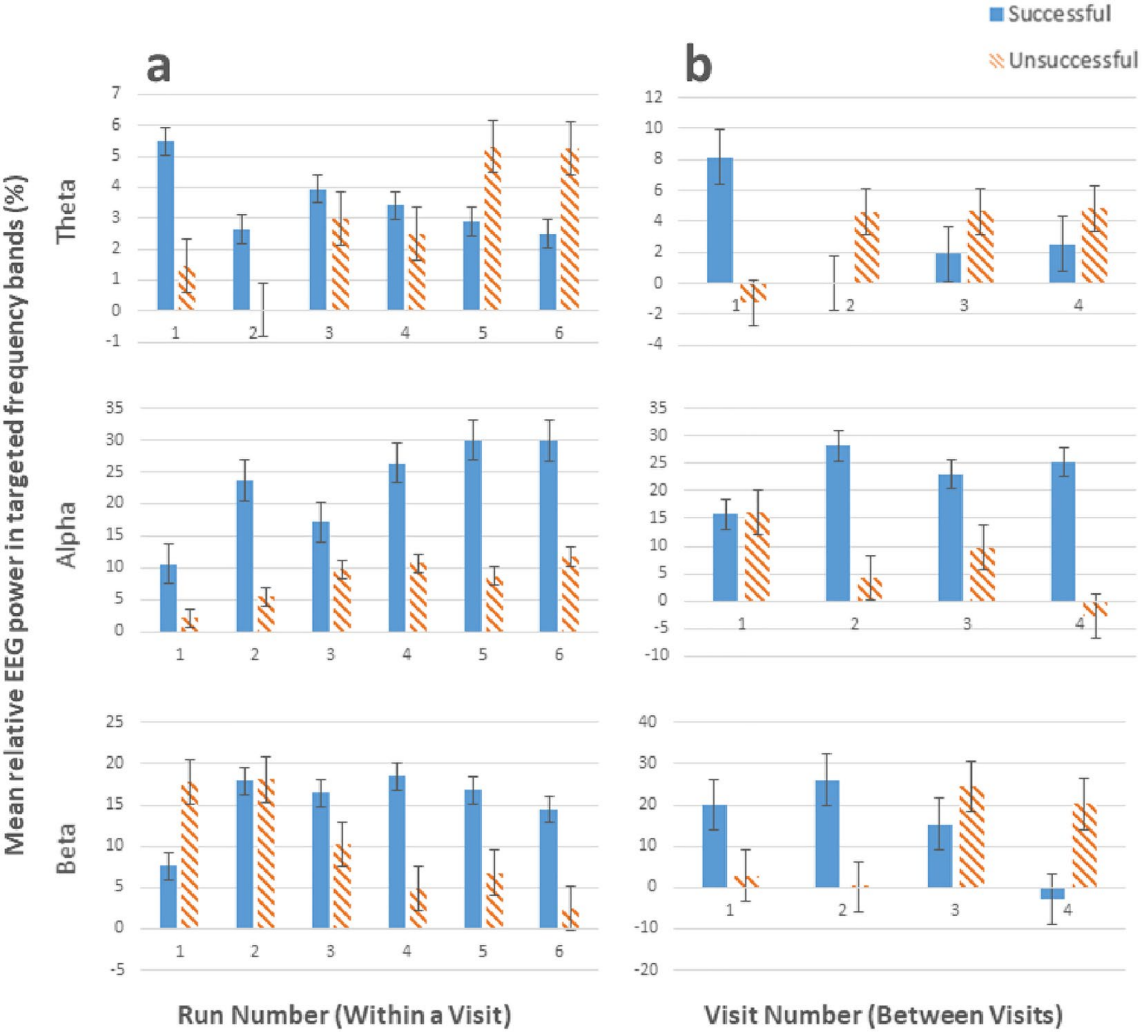
Comparisons of successful and unsuccessful performances (**a**) within a visit (i.e. between runs) and (**b**) between visits for each frequency band (theta, alpha, and beta).

**Figure 4:**
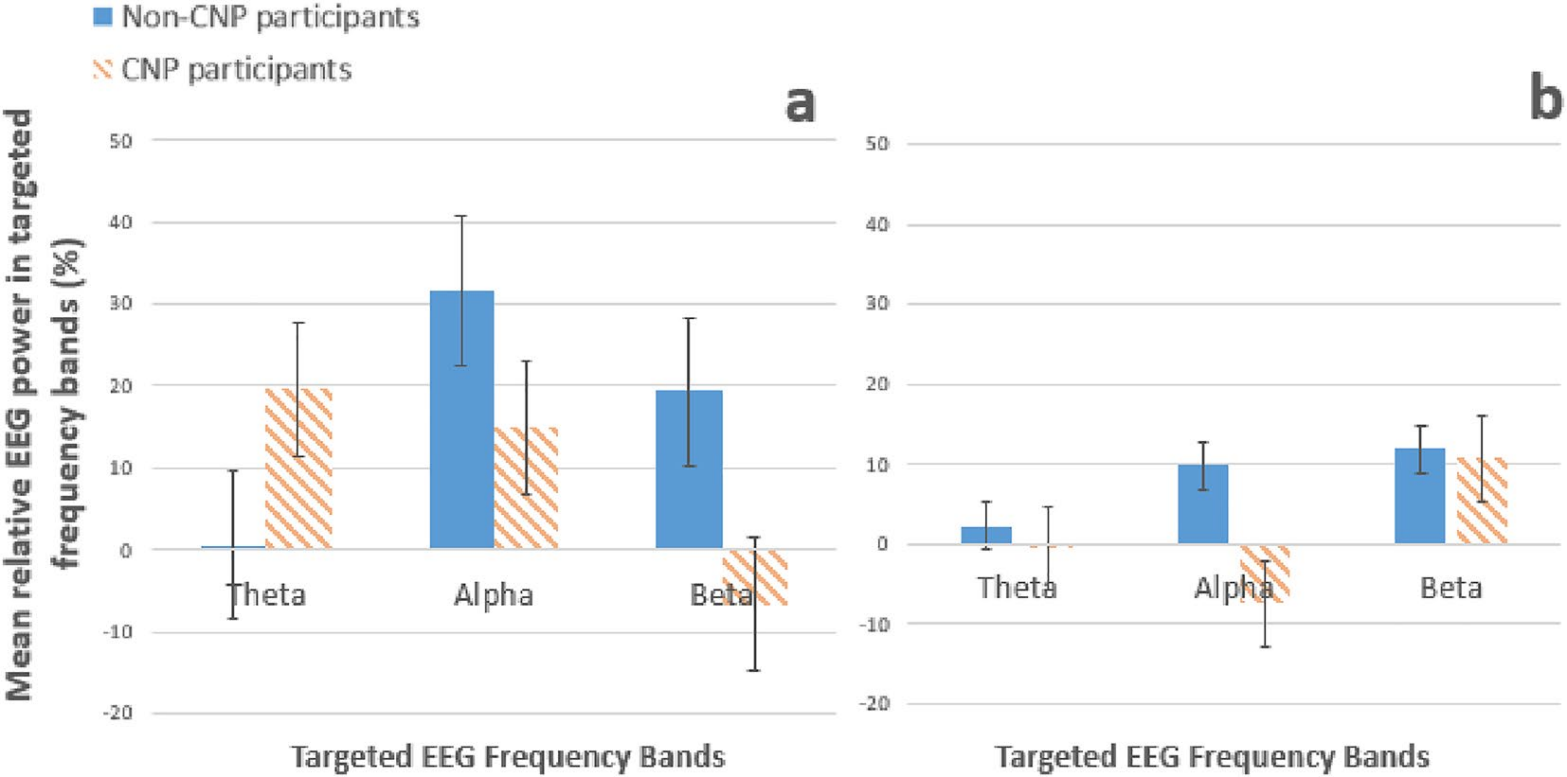
Comparisons between non-CNP participants and CNP participants categorised by (**a**) successful and (**b**) unsuccessful participants for each frequency band (theta, alpha, and beta).

Thirty-five participants were recruited: 25 were non-CNP participants (13 female; mean age = 30.96, SD = 11.19, range = 19–65) and ten were CNP participants (3 female; mean age = 51.70, SD = 10.55, range = 35–68). Participant IDs are denoted with “A” and “B” for non-CNP participants and CNP participants respectively. Table 2 displays details of CNP after SCI for each CNP participant. Fifteen participants (43%) were identified as successful at the neurofeedback task, which consisted of 10 non-CNP participants (40% of 25) and five CNP participants (50% of 10). Comparisons of successful and unsuccessful performances within a visit (i.e. between runs) and between visits for each frequency band are shown in Fig. 3. Comparisons of non-CNP and CNP participants with successful and unsuccessful performances for each frequency band are shown in Fig. 4. Not all participants completed all four neurofeedback training visits; reasons for attrition include not perceiving success at neurofeedback and non-study related events (e.g. transport to study site unavailable). Sample size for each visit is as follows: V1 = 35, V2 = 31, V3 = 27, and V4 = 25.

**Table 2:** CNP participant demographics.

ID	Years since injury	Years since pain	Pain level	ASIA injury level	Completeness
*1B*	2.5	2.5	Below-level	T12	Complete
*3B*	4	4	Both at and below-level	C4	Incomplete
*4B*	36	36	Below-level	L1	Incomplete
*5B*	48	2.3	Below-level	T6	Incomplete
*6B*	13	13	Below-level	T4	Complete
*7B*	11	4.5	Below-level	T8	Complete
*8B*	20	20	Below-level	T4	Incomplete
*9B*	4	4	Below-level	C4	Incomplete
*10B*	13	10	Both at and below-level	C6	Incomplete
*12B*	36	36	Below-level	T4	Incomplete

*CNP* central neuropathic pain, “B” denotes CNP participants.

Two CNP participants (i.e. 2B and 11B) were removed from the analysis due to excessive noise in the EEG data that prevented EEG analysis.

The comparison of neurofeedback runs in Fig. 3 shows that, unsurprisingly, participants identified as successful generally had better neurofeedback performance in theta and alpha control compared to unsuccessful participants. However, the opposite was true for beta. This is in line with the interview data, as only 8 out of 35 participants reportedly tried to control beta (7 of which were identified as unsuccessful). Figure 3 shows that successful participants improved more between visits than within a visit, indicating that more visits improve overall neurofeedback performance for successful participants. While unsuccessful participants improved their alpha within a visit; their performance was worse than successful participants. Unsuccessful participants also performed better at beta control compared to successful participants. However, Fig. 3 shows that this performance was not maintained between visits, suggesting that EEG changes within a visit for unsuccessful participants may not be due to neurofeedback learning but due to another behavioural phenomena not examined in this paper.

The descriptive statistics of success status and group category were compared and visualised in Fig. 4. The comparison in Fig. 4a reveals that non-CNP participants performed better than CNP participants in controlling alpha, while CNP participants performed better than non-CNP participants in controlling beta. However, as mentioned in the above paragraph, only one participant attempted to control beta and the remaining participants of both groups focused on alpha control. Figure 4b (showing unsuccessful participants) reveals that non-CNP participants showed better performance controlling alpha than CNP participants. The comparisons in Fig. 4 should be taken with caution due to the small sample size (n = 10) of the CNP participants.

### Neurofeedback success and mental behaviours

In the interview, participants were asked two distinct questions: (1) what they did to try and achieve the neurofeedback task and (2) how they felt during the neurofeedback training. The MB were divided into two categories based on these questions: mental strategies (a goal-directed mental action; MS) and affect (the emotional experience during neurofeedback). Thirteen MS were identified from the interview data (Table 3). Cases where participants used more than one strategy in a run are labelled “various strategies”, and cases where participants were distracted are labelled “distracted”. There were no clear differences in the prevalence of the MS between the non-CNP and CNP participants, likely due to the large variance in the MS used in both groups.

**Table 3:** List of the mental strategies identified and their descriptions.

Mental strategy	Description	Example quote
Actual movement	These mental strategies were focused on their physical body, and not imagery within the mind, where participants initiated actual movement	“[I] shifted my eyes to one direction and then the other”—22A
Auditory	Sounds derived from imagination or memory, or sounds that participants spoke/sang in their head	“I was just singing some Hindi songs [in my head]”—10B
Breathing	Participants focused on their breathing by either noticing their breathing or trying to control it	“*I did breathing, concentrated purely on breathing*”—1B
Clear mind	Clearing the mind from any thoughts to have an empty mind	“*I tried to not think of anything*”—11A
Imagination	Imagery or scenarios that the participants have not personally experienced	“*I [imagined] a leaf, growing on an oak tree… the little leaf forming the little bud, bursting [from] the tree, going through its whole cycle and then into autumn and the leaf falling off the tree*”—3B
Imagined movement	Strategies that involved imagery of moving a body part in a specific way, either from imagination or memory	“I [imagined] wiggling my toes or [stretching] the toes”—8A
Memory	Imagery or events derived from memory	“*We had a party there once, there [were] three of us sitting in the garden having a barbecue*”—16A
Moral values	Thinking about a moral value that was important to oneself	“*I was thinking about decisions and I have been thinking a lot about the tennis [match] and I was thinking that I was really pleased to have stuck to the rules of the contest*”—25A
Non-specific focus	Focus on the neurofeedback task with no verbalisation about thoughts or imagery	“*I just concentrated as hard as I could*”—4B
Numerical task	Mental strategies involving a simple numerical task	“*I just ended up trying to concentrate on doing a list of prime numbers*”—9A
Pain memory	Recalling physical pain from a past event	“*Like trying to picture like an ache going down my—in the middle of my back*”—2A
Planning	Mentally preparing for a future event	“*I think I was thinking of my essay and what I was going to do, thinking about the different things I need to [do], different materials I can use, and where I’m going to do it*”—11A
Resolving stress	Considering possible solutions to current stressful events in one's life	“*I was actually beginning to see a way through some of those issues, the issues that I was thinking about, and finding a solution to a couple of the things*”—3B

“A” and “B” denotes non-CNP and CNP participants respectively.

Table 4 shows the relationship between neurofeedback success and each MS, where success rate percentages are based on the total number of participants who used that strategy. No MS can be concluded to be better or worse for neurofeedback success as some successful strategies with a high success rate were used by a low number of participants, e.g. Pain Memory had a 100% success rate but was used only by a single (non-CNP) participant.

**Table 4:** The success rates of each Mental Strategy (MS) and the number of successful participants that used that strategy; ordered from most used to least used.

Mental strategy (MS)	No. of successful participants using MS (total n* using MS)	Success rate in %
Various strategies	9 (20)	45.00
Imagination	8 (19)	42.11
Distraction	5 (19)	26.32
Auditory	5 (18)	27.78
Imagined movement	8 (17)	47.06
Non-specific focus	5 (16)	31.25
Memory	6 (14)	42.86
Clear mind	6 (11)	54.55
Breathing	4 (10)	40.00
Actual movement	3 (5)	60.00
Numerical task	0 (4)	0.00
Planning	1 (4)	25.00
Moral values	1 (2)	50.00
Pain memory	1 (1)	100.00
Resolving stress	1 (1)	100.00

*n = no. of participants.

Affect identified from the interview analysis are shown in Table 5. Non-CNP and CNP participants shared all identified affect, with no clear differences in the prevalence of affect type**.** Table 6 displays the reported affect and their respective success rates. The number of participants reporting each affect was much larger than the numbers reporting each MS (excluding the “excited” affect). No affect can be said to be associated with neurofeedback success; however, “mentally tired” and “discontented” seem to be associated with being unsuccessful. Care needs to be taken when considering the data for the “neutral” affect: seven of the unsuccessful participants that reported this affect felt they could not accomplish the neurofeedback task no matter what they tried, and indicated that because they felt this way, they did not care about failing at the neurofeedback task. They described their affect during the remaining neurofeedback training as “neutral”. This indicates that the success rate for the “neutral” affect may be more related to some participants’ effort and not the affect itself. These participants were kept in the analysis because they continued to attempt the neurofeedback task.

**Table 5:** Success rates of affect induced by mental strategies.

Affect	No. of successful participants reporting affect (total number of participants reporting affect)	Success rate (%)
Discontent	13 (30)	43.33
Excited	2 (4)	50.00
Happy	10 (18)	55.56
Mentally tired	8 (22)	36.36
Neutral	11 (28)	39.29
Relaxed	12 (24)	50.00

**Table 6:** Run frequencies between participant group and affect, and group and mental strategy.

	Run count (%) (non-CNP)	Run count (%) (CNP)
**Affect**		
Excited	7 (2.98)	29 (37.66)
Happy	60 (25.53)	0 (0)
Neutral	32 (13.62)	22 (28.57)
Discontent	35 (14.89)	2 (2.6)
Mentally tired	34 (14.47)	3 (3.9)
Relaxed	67 (28.51)	21 (27.27)
Total	235 (100)	77 (100)
**MS**		
Various strategies	15 (6.38)	38 (49.35)
Non-specific focus	6 (2.55)	26 (33.77)
Imagined movement	73 (31.06)	5 (6.49)
Memory	33 (14.04)	0 (0)
Imagination	33 (14.04)	1 (1.30)
Clear mind	34 (14.47)	2 (2.60)
Resolving stress	0 (0)	2 (2.60)
Auditory	10 (4.26)	0 (0)
Breathing	11 (4.68)	1 (1.30)
Actual movement	4 (1.70)	0 (0)
Moral values	3 (1.28)	0 (0)
Pain memory	2 (0.85)	0 (0)
Planning	2 (0.85)	0 (0)
Distraction	9 (3.83)	2 (2.60)
Total	235 (100)	77 (100)

*Indicates significant p-value based on adjusted α.

*MS* mental strategies, *CNP* central neuropathic pain.

### Comparison of mental behaviours between successful non-CNP and CNP participants

The descriptive statistics (i.e. run count and percentage) in Table 6 below shows that CNP participants did not report the Happy affect, and instead reported more Neutral or Excited affects compared to non-CNP participants. Non-CNP participants reported more negative affect than (Discontent and Mentally Tired) than CNP participants. Table 6 also shows that CNP participants, compared to non-CNP participants, preferred using Various Strategies and Non-Specific Focus, and did not use Memory. Non-CNP participants preferred using Imagined Movement and Imagination compared to CNP participants. However, this analysis should be taken with caution due to the limited CNP participant sample size and attrition rate (see Table 1 in the supplementary materials file “SM2—Extra Tables”).

### The association between mental strategies and affect

MS were matched with their respective affect for each neurofeedback training run to explore the association between them. After removal of inadequate runs and all instances where multiple strategies were used, a total of 551 runs were examined. Table 7 summarises the affect reported during each run of each identified mental strategy. Some relationships are unsurprising, for example, Clearing Mind was mostly associated with the Relaxed affect. However, positive mental strategies were associated with negative affect, which appeared from interview data to be related to finding the neurofeedback task itself boring or frustrating or being disappointed with their perceived success rather than a consequence of the mental strategies applied.

“[That run] was worse than normal, and then I felt a bit down from [my poor performance]”—10A (whilst using Clear Mind).

“*I may have just [been] bored looking at the screen*”—25A (whilst using Positive Memory).

**Table 7:** Matching mental strategy with affect.

	No. of instances of each identified affect						
	Discontent	Excited	Happy	Mentally tired	Neutral	Relaxed	Total (%)
**MS**							
Clear mind	7	0	2	6	5	33*	53 (9.62)
Actual movement	0	0	0	0	3*	3*	6 (1.09)
Imagination	20	2	13	14	24*	14	87 (15.79)
Non-specific focus	32	0	0	9	37*	19	97 (17.60)
Numerical task	1	0	0	3	8*	2	14 (2.54)
Breathing	1	0	0	4	11	15*	31 (5.63)
Memory	1	4	23*	7	8	14	57 (10.34)
Auditory	14	0	6	14	33*	5	72 (13.07)
Moral values	1	0	4*	3	1	2	11 (2.00)
Pain memory	0	0	2*	0	0	0	2 (0.36)
Planning	2	1	4*	0	2	0	9 (1.63)
Resolving stress	1*	0	1*	0	0	0	2 (0.36)
Imagined movement	11	8	30	14	33*	14	110 (19.96)
Total (%)	91 (16.52)	15 (2.72)	85 (15.43)	74 (13.43)	165 (29.95)	121 (21.96)	551

*The most associated affect for each mental strategy.

*MS* No. of instances of each identified mental strategy.

Pain Memory was only associated with the Happy affect. The sole participant who used this mental strategy reported that the pain was associated with a sport that they enjoyed (“… [sports] is a positive thing for me”—2A). The pain was an accepted part of playing that sport, thus the Pain Memory induced a positive emotion.

“*I have the mind-set that when you’re training for [the sport], you go through times when you just hurt a lot… You get used to it… it’s not a negative thing*”—2A.

### Questionnaire analysis of general learning factors

**Figure 5:**
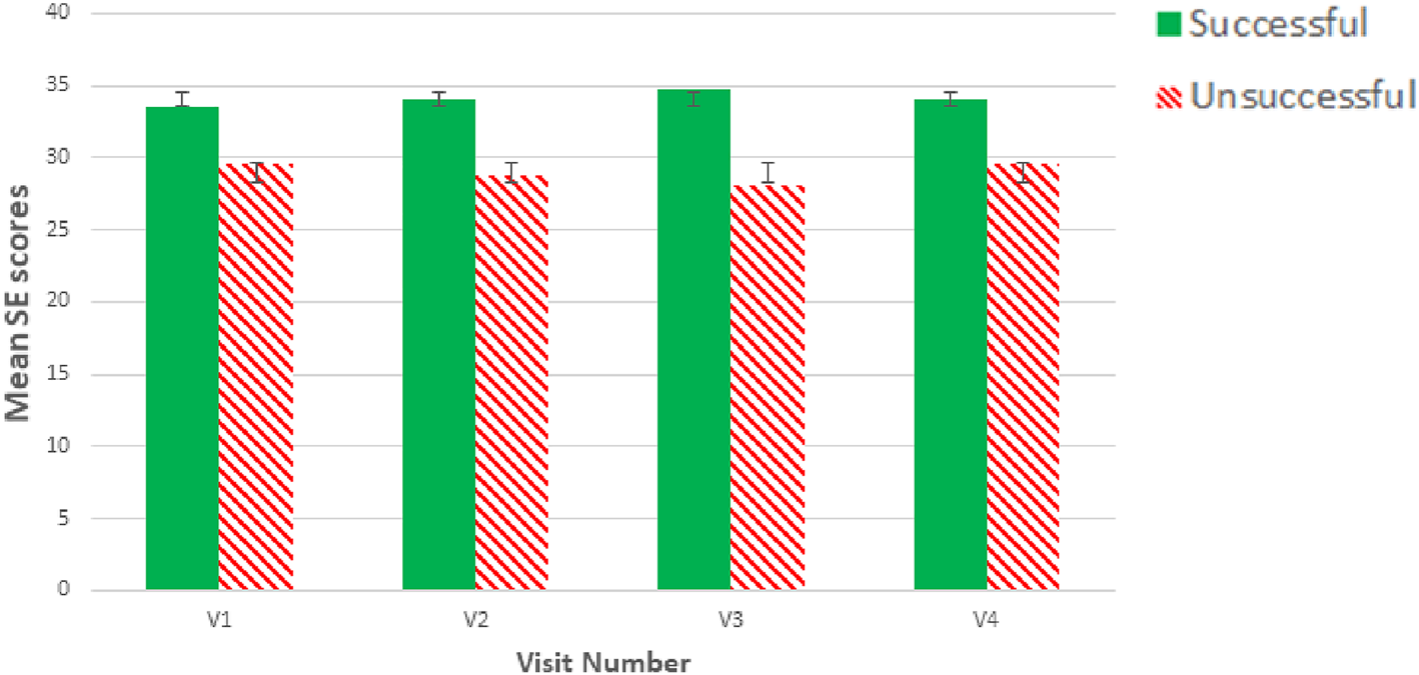
Bar graph depicting correlation between SE and neurofeedback success for all visits.

As a reminder to the reader, fifteen participants were identified as successful at neurofeedback, of whom 10 were non-CNP participants and five were CNP participants. Pearson’s point–biserial correlation coefficient was used to assess the relationship between the scores of general learning factor questionnaires (SE, task load, motivation, and LoC) and the aforementioned success rate; this was conducted for each visit (descriptive statistics for each measure are shown in Table 1 in the supplementary material file “SM2—Extra Tables”). Correlation for LoC was only conducted for non-CNP participants as CNP participants completed a different LoC questionnaire and there were only 10 CNP participants. There was a statistically significant, moderate correlation between SE and success for all visits (V1: r = − 0.430, p = 0.010, n = 35; V2: r = − 0.505, p = 0.004, n = 31; V3: r = − 0.587, p = 0.001, n = 27; V4: r = − 0.461, p = 0.020, n = 25), where higher SE scores were associated with success (Fig. 5). All other correlations were low (r < 0.300) and non-significant (p > 0.05). Table 1 in the supplementary material file “SM2—Extra Tables” displays the descriptive statistics for all questionnaire scores between successful and unsuccessful participants per visit.

## Discussion

This study aimed to address the following questions: (1) “what MB do participants use to succeed at neurofeedback?” and (2) “what is the relationship between general learning factors (i.e. LoC, SE, motivation, and difficulty) and neurofeedback performance?” No distinct relationship was found between MB (i.e. including both mental actions and affect) and neurofeedback success. However, negative affect (i.e. Mentally Tired and Discontented) were somewhat linked with an unsuccessful neurofeedback performance. SE was the only general learning factor to be associated with neurofeedback performance, where SE moderately increased with successful performance. CNP participants reported being Neutral or Excited, and did not report the Happy affect compared to non-CNP participants. Yet, non-CNP participants reported more negative affect compared to CNP participants. This contradicts other research examining MS; for example, Nan et al.^21^ and Hardman et al.^43^ found that happy MS were linked with raising alpha power and regulating interhemispheric frontal asymmetry respectively. CNP participants used more Various Strategies and Non-Specific Focus MS and did not use Memory, while non-CNP participants used Imagined Movement and Imagination. While this indicates that CNP participants used distinct MS compared to non-CNP participants for neurofeedback success, this finding must be taken with caution due to the difference in sample sizes between the participant groups. Additionally, considerably more CNP participants dropped out of the study than non-CNP participants, likely due to the greater burden of study participation for the CNP participants. This may be the reason why the affect-related findings contradict the other research mentioned here. Larger and more balanced sample sizes would be required to confirm the differences between the groups observed here and examine whether neurofeedback guidance needs to be tailored specifically for the context of pain for those with CNP after SCI.

No individual MS (i.e., mental actions; excluding affect) were clearly linked with success; this is likely due to the use of varied MS across participants. Nan et al.^21^ and Kober et al.^20^ also asked participants to identify their own MS. Nan et al.^21^ (regulating the alpha frequency) found that 61.29% of MS used were positive, 33.87% were neutral, and 4.84% were negative; Kober et al.^20^, (regulating SMR) found seven categories of MS, where usage varied from 45% in “concentration” to 5% in “breathing”. Although these studies infer a relationship between MS and neurofeedback success, the use of varied MS makes it difficult to draw confident conclusions. It is possible that MS do not have a crucial relationship with neurofeedback success. This is further supported by Siniatchkin et al.^44^, who reversed neurofeedback conditions (i.e. from increasing slow cortical potentials (SCP) to decreasing SCP) halfway through neurofeedback training without informing participants. Participants initially performed worse after the reversal but were able to improve their performance after realisation of the reversal. Yet, participants reported using the same MS throughout the entire neurofeedback training. Siniatchkin et al.^44^ concluded that knowledge of the neurofeedback conditions were more important than using the “correct” MS.

The negative affect of Mentally Tired and Discontented were linked with being unsuccessful at neurofeedback. It may be that unsuccessful performances frustrate participants, resulting in a negative affect. However, it may also indicate that inducing a negative affect may reduce chances of success at the neurofeedback task. Nijboer et al.^18^ found that a more positive mood was linked with better performance at their neurofeedback task (changing the amplitude of SMR). Yet, in a later study, Nijboer et al.^45^ found no influence of mood on neurofeedback performance [influencing SMR and ERP (event-related potential)]. This conflicting evidence may be due to the use of different neurofeedback protocols. Further research is needed to understand how affect influences (and influenced by) neurofeedback performance, such as examining the type of affect as well as the source of the affect and its relationship to different neurofeedback protocols.

This study separates MS from affect whereas this distinction is not reported by other studies, such as Kober et al.^20^ who identified their Relax theme as a MS, while this paper identified it as an affect. The importance in this distinction is reflected in the current finding that a MS may not induce the expected affect. Additionally, the affect may not be induced by the MS but by another source, such as perception of neurofeedback performance or an unrelated event (e.g. noise distraction). Distinguishing MS from affect may also aid in understanding qualities that improve neurofeedback performance.

SE was the only general learning factor that had a significant, positive correlation with success. This indicates that those who have lower SE may give up on accomplishing the neurofeedback task earlier than those who have higher SE, thus reducing the likelihood of finding an appropriate MB that improves neurofeedback performance. Current literature examines how neurofeedback influences SE as a clinical outcome^46,47^, and concludes that neurofeedback has the potential to improve SE. However, there is no research that examines SE as a predictor of neurofeedback learning. The current SE finding has potential to be used to develop a standard for verbal guidance; SE’s positive correlation with neurofeedback success suggests that boosting users’ SE and self-appraisal can influence neurofeedback performance. Given that SE appears to have a relationship with neurofeedback success and that there were no obvious links between MB and success, it may be that the instructions and feedback given to support participants SE may be very important for neurofeedback success. Further examination is needed to confirm the relationship between SE and neurofeedback success, and to identify techniques that may enhance participant’s neurofeedback SE.

The lack of correlation between LoC and success aligns with previous research, as only technology-related LoC has been found to have a relationship with neurofeedback success^48,49^ and this was not measured in this study. It is unclear why the difficulty (i.e. task load) yielded a non-significant, low correlation with neurofeedback success. Previous studies monitored task load, or aspects of task load such as effort^41^, and found that adapting the neurofeedback task accordingly increased the likelihood of neurofeedback success. This suggests that an adaptable neurofeedback software may be more important than the perceived difficulty. Similarly, it is unclear why motivation also yielded a non-significant, low correlation. All participants had moderately high motivation to complete the study, which may be why motivation was not found to be associated with neurofeedback success. This may be an example of recruitment bias, where individuals who volunteer for research studies are likely to provide different results than those who do not volunteer for research studies^50^. Although Kadosh and Staunton^16^ also state that motivation tends to be high due to recruitment bias, they found that five out of eight studies examining motivation established it as a positive influence on neurofeedback performance^16^. However, studies which had larger sample sizes^51,52^ found that motivation does not influence neurofeedback performance, concurring with the motivation finding in this paper. This suggests that motivation may only reflect participants’ willingness to engage with the task and not their neurofeedback ability.

There are several limitations to this study. Firstly, the number of visits (4) were small and may have limited the number of successful participants; more visits may have resulted in a higher success rate. Furthermore, the number of neurofeedback training visits varied amongst participants due to drop-outs. In addition, not only did participants report more than one MS and affect, the number of participants that used each MS and affect varied greatly and therefore introduces the issue of over-representation of certain MS/affect over others. This prevented the use of statistical testing, such as chi-squared tests, to further explore any relationships between variables. However, the primary aim of this study was to qualitatively explore neurofeedback learning, as a precursor for more focused hypothesis testing studies. The benefit of the current study is the qualitative approach, which provides depth of information from each participant. The use of separate LoC measure for non-CNP participants and participants with CNP after SCI, chosen to explore their different circumstances, decreased the statistical power of the correlation for CNP participants due to their small sample size. However, LoC was not found to significantly correlate with neurofeedback success in the non-CNP participant group, which suggests that LoC may also not correlate with neurofeedback success in CNP participants. The findings in this study may only be applicable to the current neurofeedback protocol, where findings may change with self-regulation of different features of EEG activity. Finally, although this study did not aim to examine the neurofeedback protocol’s effectiveness for pain relief, it may have been useful to monitor pain to better understand the neurofeedback protocol and its learning process. Future research should use a randomised-controlled design with equal and larger samples sizes to understand the relationship between neurofeedback-induced pain relief and MB.

This paper has indicated the potential involvement of SE in neurofeedback learning and its implication for the impact of the instructions on how to perform neurofeedback. It has also emphasised the complexity of behaviours involved in neurofeedback learning, such as the variety of MB involved and the potential influence of negative affect. Future research should examine the relationship between SE and neurofeedback success by providing SE-based guidance and comparing results to a control group. This may be especially important in the CNP after SCI population, as SE is consistently reported lower in this population^53,54^. Future research should also investigate the influence of the affect by inducing negative, positive, and neutral affect during neurofeedback training in three separate groups, and comparing results to a control group. Finally, it is crucial to separate MB into MS and affect to better understand the nuances of neurofeedback learning.

## Supplementary Information

Supplementary Information 1.

Supplementary Information 2.

Supplementary Information 3.

## Data Availability

Data available as a supplementary Excel file.
