# Supplementary material for: The importance of self-efficacy and negative affect for neurofeedback success for central neuropathic pain after a spinal cord injury
Source: Sci Rep. 2022 Jun 29;12:10949. doi: 10.1038/s41598-022-15213-7 (PMC9243249; doi:10.1038/s41598-022-15213-7)
Supplement: Supplementary file 1 — Supplementary Information 1. [file 41598_2022_15213_MOESM1_ESM.docx]

**Title**

The importance of self-efficacy and negative affects for neurofeedback success for central neuropathic pain after a spinal cord injury

**Authors**

Krithika Anil^1,2^* (Orcid ID: 0000-0002-8027-1665), Sara Demain^2^, Jane Burridge^3^, David Simpson^1^, Julian Taylor^4,5^, Imogen Cotter^6^, Aleksandra Vuckovic^7^

1 Faculty of Engineering and Physical Sciences, University of Southampton, Southampton, UK

2 Faculty of Health and Human Sciences, University of Plymouth, Plymouth, UK

3 Faculty of Health Sciences, University of Southampton, Southampton, UK

4 Sensorimotor Function Group, Hospital Nacional de Parapléjicos, SESCAM, Toledo, Spain

5 Harris Manchester College, University of Oxford, Oxford, UK

6 Department of Clinical Psychology, National Spinal Injuries Centre, Stoke Mandeville Hospital, Aylesbury, UK

7 Department of Biomedical Engineering, School of Engineering, University of Glasgow, Glasgow, UK

*Corresponding author: Krithika Anil

Contact email: krithika.anil@plymouth.ac.uk

**Interview Analysis – Supplementary Information 1**

This document contains information related to the interview analysis, and begins with a detailed description of the interview analysis. The following tables are also provided for further information:

- Table A1 – Interview schedule followed after each neurofeedback visit
- Table A2 – Categorisation of initial codes into themes regarding mental strategies
- Table A3 – Categorisation of initial codes into themes regarding affects
- Table A4 – COREQ (COnsolidated criteria for REporting Qualitative research) Checklist

**Detailed Interview Analysis**

Interview data were analysed by KA using thematic analysis and a framework model as described by Gale et al (Gale, Heath, Cameron, Rashid, & Redwood, 2013). See Table A1 below for the interview schedule.

Table A1 Interview schedule followed after each neurofeedback visit

| To gain information on what mental strategies were used |
| --- |
| 1. Can you please describe to me, for each neurofeedback training run, what you did to try to make the bars green? |
| 1. Did you try anything else? |
| To understand the quality of mental strategy used |
| 1. Can you tell me more about [insert strategy used by individual]? |
| 1. Can you tell me why you used [insert strategy used]? |
| 1. How did you feel when you tried [insert strategy used]? |
| 1. How did you know that [insert strategy used] was working? |
| 1. How did you know that [insert strategy used] was not working? |
| 1. How distracted were you when you used [insert strategy used]? |
| 1. How did you deal with the distraction? |
| Question 1 was asked first. Questions 2 to 11 were asked for each strategy that participants mentioned. Follow-up questions were asked to gain more details of the mentioned mental strategy. |

Interview transcripts of all participants with CNP and six able-bodied participants were selected to develop an initial framework. All interviews from participants with CNP were analysed for the initial framework as the neurofeedback protocol was designed for these individuals (Hassan, Fraser, Conway, Allan, & Vuckovic, 2015). Interviews of six able-bodied participants were randomly selected (via random number generation in Microsoft Excel) for the initial framework as saturation of data was expected to occur after analysis of eighteen sets of interview data (twelve participants with CNP and six able-bodied participants (Guest, Bunce, & Johnson, 2006; Saunders et al., 2018)). Stage 1 of the framework analysis (Gale et al., 2013) involved transcribing verbatim the audio-recorded interviews for all participants. Stage 2 involved familiarisation with the interview content by re-reading transcripts and re-listening to the recorded audio. The remaining stages were conducted using the Nvivo software to assist in managing the data. Stage 3 involved coding any words, phrases, or paragraphs that related to the mental strategies used during the neurofeedback. Stage 4 involved organising similar codes into overarching themes to construct the initial framework. Themes were constructed based on the codes relationship to EEG activity; for example, motor imagery is seen as a distinct mental activity that changes EEG activity in a specific way (further detailed in the results section). Stage 5 involved coding the remaining transcripts by applying the themes from the initial framework. Any new themes found during this stage were added to the framework, and all previously coded transcripts were re-analysed with the updated framework. Stage 6 involved charting the themes to create the final framework, which was interpreted for the concluding stage of analysis. See Tables A2 and A3 below for summary of code categorisation.

An independent qualitative researcher re-coded interviews from six randomly selected participants according to the final framework. The independent researcher’s codes were checked against those codes from the first researcher (KA), any disagreements were discussed and coding labels refined and agreed. This study was reported using the COREQ criteria of reporting qualitative research (Booth et al., 2014) (see Table A4 at the end of this document).

Table A2 Categorisation of initial codes into themes regarding mental strategies

| **Initial Codes** | **Grouping Description** | **Themes** |
| --- | --- | --- |
| - Actually moving body part (not just in mind) | Actual Movement | These mental strategies were focused on their physical body, and not imagery within the mind, where participants initiated actual movement |
| - Chanting green - Chanting up/down - Chanting happy - Ordering bars to become green - Imagining a sound from nature - Memory of a pleasant sound - Reciting poem/prose - Recalling a song - Spelling out words | Auditory | Sounds derived from imagination or memory, or sounds that participants spoke/sang in their head |
| - Focusing on breathing - Controlling breathing | Breathing | Participants focused on their breathing by either noticing their breathing or trying to control it |
| - Emptying mind of thoughts - Not thinking of anything | Clear Mind | Participants were trying to clear their mind if thoughts |
| - Imagining calm scene - Imagining green object - Imagining green scene - Doing something to the bars | Imagination | Imagery that the participant has not personally experienced, where the mental strategies were derived from watching someone else (e.g. a person on TV) or a description they have come across (e.g. from a book) |
| - Sexual memory - Sports memory - Imagining physical movements - Imagining doing a sport - Imagining a physical sensation | Imagined Movement | Strategies that involved imagery of moving a body part in a specific way, either from imagination or memory |
| - Memory of a loved-one - Memory of a pleasant sensation - Videogame memory - Memory of driving a familiar route - Memory of a shopping route - Memory with green | Memory | Mental strategies that involved imagery derived from memory, not from imagination |
| - Thinking about a good decision - Thinking about an important personal trait | Moral Values | Participants reported that their decision/trait reflected a moral value important to them |
| - "Just concentrating" | Non-Specific Focus | Participants only reported concentrating on the neurofeedback task without further descriptions of using imagery or other thoughts |
| - Counting - Maths multiplication tables - Listing prime numbers - Counting number of times bar went green | Numerical Task | Mental strategies involving a simple numerical task |
| - Painful sensation | Pain Memory | Memory of painful sensation |
| - Planning out a meal - Planning out coursework prep - Planning future event with family | Planning | Participants were thinking of the things they needed to do in order to complete a future task |
| - Resolving personal stress | Resolving Stress | Participant reported finding possible solutions to their current personal issues that were causing them stress |

Table A3 Categorisation of initial codes into themes regarding affects

| **Initial Codes** | **Grouping Description** | **Themes** |
| --- | --- | --- |
| - Bored - Disappointed - Frustrated - Uselessness - Self-blame | Participants were unsatisfied with their neurofeedback performance resulting in various negative feelings | *Discontent* |
| - Excited | Some mental strategies or the novelty of the neurofeedback induced feelings of excitement | *Excited* |
| - Happy - Pleasant - Positive | Positive mental strategies or satisfactory neurofeedback performance produced feelings of happiness | *Happy* |
| - Mentally tiring - Mentally straining - Sleepy | Participants reported becoming mentally tired after intense concentrating or concentrating for too long on the neurofeedback task | *Mentally Tired* |
| - Neutral - Not positive or negative | Participants described feeling “neutral”, nothing in particular, either due to a neutral mental strategy or due to not caring about accomplishing the neurofeedback task | *Neutral* |
| - Relaxed - Calm - At ease | Calming mental strategies induced feelings of relaxation or ease | *Relaxed* |

Table A4 COREQ (COnsolidated criteria for REporting Qualitative research) Checklist

| **Topic** | **Item** | **Guide Questions/Description** | **Responses** |
| --- | --- | --- | --- |
| **Domain 1: Research team and reflexivity** | | | |
| *Personal characteristics* | | | |
| Interviewer/facilitator | 1 | Which author/s conducted the interview or focus group? | KA |
| Credentials | 2 | What were the researcher’s credentials? E.g. PhD, MD | BSc (Hons), MSc, PhD |
| Occupation | 3 | What was their occupation at the time of the study? | KA was a PhD candidate at the time of the study (PhD now completed) |
| Gender | 4 | Was the researcher male or female? | Female |
| Experience and training | 5 | What experience or training did the researcher have? | KA completed modules in qualitative and quantitative methods during their BSc and MSc. Further methodological training was completed during the PhD. KA also had experience working as a research assistant on a project related to the management of general chronic pain. |
| *Relationship with participants* | | |  |
| Relationship established | 6 | Was a relationship established prior to study commencement? | No relationship was established with patient participants prior to the study commencement. The first five recruited able-bodied participants were colleagues of KA. |
| Participant knowledge of the interviewer | 7 | What did the participants know about the researcher? E.g. personal goals, reasons for doing the research | During recruitment, KA explained the study aim and procedure. Participant colleagues of KA knew none of KA’s personal or professional goals as KA had little contact with these colleagues prior to the study. |
| Interviewer characteristics | 8 | What characteristics were reported about the interviewer/facilitator? e.g. Bias, assumptions, reasons and interests in the research topic | KA was the lead researcher and had an academic interest in the study. No assumptions were made by KA. Bias was reduced by using an independent qualitative researcher to validate the data analysis. |
| **Domain 2: Study design** | | | |
| *Theoretical framework* | | | |
| Methodological orientation and Theory | 9 | What methodological orientation was stated to underpin the study? e.g. grounded theory, discourse analysis, ethnography, phenomenology, content analysis | This study was part of a mixed-methods design including qualitative and quantitate measures. Thematic analysis and a framework model was used for analysis. |
| *Participant selection* | | |  |
| Sampling | 10 | How were participants selected? e.g. purposive, convenience, consecutive, snowball | Convenience sampling was used for recruitment. |
| Method of approach | 11 | How were participants approached? e.g. face-to-face, telephone, mail, email | Able-bodied participants were approached face-to-face. Able-bodied and patient participants were also recruited using posters, and could volunteer for the study by contacting KA using the email or phone number listed on the posters. |
| Sample size | 12 | How many participants were in the study? | Thirty-nine initial participants (twenty-seven able-bodied and twelve patient participants), reduced to thirty-five participants (twenty-five able-bodied and ten patient participants) after four participants were removed due to noisy EEG data.. |
| Non-participation | 13 | How many people refused to participate or dropped out? Reasons? | No participants refused to participate. Ten participants (two able-bodied participants and eight patients) dropped out due to perception of poor neurofeedback performance or non-study related events (unavailable transport, feeling unwell, or an unexpected personal event). |
| *Setting* | | |  |
| Setting of data collection | 14 | Where was the data collected? e.g. home, clinic, workplace | Interviews were carried out at a designated study site at the university or the hospital. |
| Presence of non-participants | 15 | Was anyone else present besides the participants and researchers? | During two interviews, a supervisor observed the study procedure. No third person was present at all other study procedures. |
| Description of sample | 16 | What are the important characteristics of the sample? e.g. demographic data, date | All participants were aged eighteen or over. Able-bodied participants had no chronic pain or neurological conditions. Patient participants had chronic neuropathic pain after a spinal cord injury, and had no other neurological conditions. |
| *Data collection* | | |  |
| Interview guide | 17 | Were questions, prompts, guides provided by the authors? Was it pilot tested? | Interview questions were devised by KA and SD (last author, an experienced qualitative researcher and supervisor), and discussed within the research team. The interview questions were piloted with KA’s colleagues who did not participate in the main study. |
| Repeat interviews | 18 | Were repeat interviews carried out? If yes, how many? | Four interviews on separate occasions were conducted per participant. |
| Audio/visual recording | 19 | Did the research use audio or visual recording to collect the data? | All interviews were audio-recorded. |
| Field notes | 20 | Were field notes made during and/or after the interview or focus group? | Brief field notes were made during the interview, and reflections were noted after each interview. |
| Duration | 21 | What was the duration of the interviews or focus group? | Interviews ranged from six minutes to forty-five minutes, but were generally fifteen minutes. |
| Data saturation | 22 | Was data saturation discussed? | Data saturation was discussed between KA and SD, and were confident saturation had been achieved within the recruited sample. |
| Transcripts returned | 23 | Were transcripts returned to participants for comment and/or correction? | Transcripts were not returned to participants. |
| **Domain 3: analysis and findings** | | | |
| *Data analysis* | | | |
| Number of data coders | 24 | How many data coders coded the data? | KA coded all the interview data. An independent qualitative researcher coded transcripts of six randomly chosen participants. |
| Description of the coding tree | 25 | Did authors provide a description of the coding tree? | Coding is summarised in the appendix, detailing the categorisations of initial codes into the main theme. |
| Derivation of themes | 26 | Were themes identified in advance or derived from the data? | Themes were derived from the data and cognitive-neuro literature. |
| Software | 27 | What software, if applicable, was used to manage the data? | Nvivo, version 12. |
| Participant checking | 28 | Did participants provide feedback on the findings? | No. |
| *Reporting* | | |  |
| Quotations presented | 29 | Were participant quotations presented to illustrate the themes/findings? Was each quotation identified? e.g. participant number | Quotations are provided throughout the results of the paper to illustrate each theme. Quotation are identified by the participant number, and a ‘A’ for able-bodied participants and a ‘B’ for participants with CNP. |
| Data and findings consistent | 30 | Was there consistency between the data presented and the findings? | The themes were derived from the data and existing cognitive-neuro literature, therefore there is consistency between the data presented and the findings. |
| Clarity of major themes | 31 | Were major themes clearly presented in the findings? | The major themes are clearly presented within the paper. |
| Clarity of minor themes | 32 | Is there a description of diverse cases or discussion of minor themes? | Diverse cases and minor themes are presented in the results. |

**References**

Booth, A., Hannes, K., Harden, A., Noyes, J., Harris, J., & Tong, A. (2014). COREQ (Consolidated Criteria for Reporting Qualitative Studies). In (pp. 214-226).

Gale, N. K., Heath, G., Cameron, E., Rashid, S., & Redwood, S. (2013). Using the framework method for the analysis of qualitative data in multi-disciplinary health research. *BMC Medical Research Methodology, 13*, 117-117. doi:10.1186/1471-2288-13-117

Guest, G., Bunce, A., & Johnson, L. (2006). How many interviews are enough? An experiment with data saturation and variability. *Field methods, 18*(1), 59-82.

Hassan, M. A., Fraser, M., Conway, B. A., Allan, D. B., & Vuckovic, A. (2015). The mechanism of neurofeedback training for treatment of central neuropathic pain in paraplegia: a pilot study. *BMC Neurol, 15*, 200. doi:10.1186/s12883-015-0445-7

Saunders, B., Sim, J., Kingstone, T., Baker, S., Waterfield, J., Bartlam, B., . . . Jinks, C. (2018). Saturation in qualitative research: exploring its conceptualization and operationalization. *Quality & quantity, 52*(4), 1893-1907.
