# Supplementary material for: The importance of self-efficacy and negative affect for neurofeedback success for central neuropathic pain after a spinal cord injury
Source: Sci Rep. 2022 Jun 29;12:10949. doi: 10.1038/s41598-022-15213-7 (PMC9243249; doi:10.1038/s41598-022-15213-7)
Supplement: Supplementary file 2 — Supplementary Information 2. [file 41598_2022_15213_MOESM2_ESM.docx]

**Title**

The importance of self-efficacy and negative affects for neurofeedback success for central neuropathic pain after a spinal cord injury

**Authors**

Krithika Anil^1,2^* (Orcid ID: 0000-0002-8027-1665), Sara Demain^2^, Jane Burridge^3^, David Simpson^1^, Julian Taylor^4,5^, Imogen Cotter^6^, Aleksandra Vuckovic^7^

1 Faculty of Engineering and Physical Sciences, University of Southampton, Southampton, UK

2 Faculty of Health and Human Sciences, University of Plymouth, Plymouth, UK

3 Faculty of Health Sciences, University of Southampton, Southampton, UK

4 Sensorimotor Function Group, Hospital Nacional de Parapléjicos, SESCAM, Toledo, Spain

5 Harris Manchester College, University of Oxford, Oxford, UK

6 Department of Clinical Psychology, National Spinal Injuries Centre, Stoke Mandeville Hospital, Aylesbury, UK

7 Department of Biomedical Engineering, School of Engineering, University of Glasgow, Glasgow, UK

*Corresponding author: Krithika Anil

Contact email: krithika.anil@plymouth.ac.uk

**Extra Tables – Supplementary Information 2**

Table 1 Mean and standard deviations of questionnaire scores for each visit

|  | **Visit 1 (N = 35)** | | **Visit 2 (N = 31)** | | **Visit 3 (N = 27)** | | **Visit 4 (N = 25)** | |
| --- | --- | --- | --- | --- | --- | --- | --- | --- |
| ***SE*** | *Mean* | *SD* | *Mean* | *SD* | *Mean* | *SD* | *Mean* | *SD* |
| *Successful* | 33.53 | 3.44 | 34.07 | 4.14 | 34.69 | 3.99 | 34.00 | 4.53 |
| *Unsuccessful* | 29.55 | 4.77 | 28.82 | 4.97 | 28.07 | 5.34 | 29.54 | 4.41 |
|  | **Visit 1 (N = 35)** | | **Visit 2 (N = 31)** | | **Visit 3 (N = 27)** | | **Visit 4 (N = 25)** | |
| ***Task Load*** | *Mean* | *SD* | *Mean* | *SD* | *Mean* | *SD* | *Mean* | *SD* |
| *Successful* | 58.11 | 17.87 | 56.90 | 18.51 | 50.67 | 13.86 | 43.42 | 14.35 |
| *Unsuccessful* | 60.70 | 17.98 | 56.18 | 13.56 | 58.31 | 19.87 | 55.49 | 15.55 |
|  | **Visit 1 (N = 35)** | | **Visit 2 (N = 31)** | | **Visit 3 (N = 27)** | | **Visit 4 (N = 25)** | |
| ***Motivation*** | *Mean* | *SD* | *Mean* | *SD* | *Mean* | *SD* | *Mean* | *SD* |
| *Successful* | 7.33 | 1.35 | 7.43 | 1.79 | 7.23 | 1.64 | 6.67 | 1.97 |
| *Unsuccessful* | 7.50 | 1.67 | 6.76 | 2.02 | 6.21 | 2.64 | 6.54 | 2.03 |
|  | **Visit 1 (N = 25)** | | **Visit 2 (N = 23)** | | **Visit 3 (N = 22)** | | **Visit 4 (N = 22)** | |
| ***^AB^LoC – Internality*** | *Mean* | *SD* | *Mean* | *SD* | *Mean* | *SD* | *Mean* | *SD* |
| *Successful* | 34.00 | 4.32 | 36.70 | 4.79 | 36.00 | 4.57 | 36.30 | 5.23 |
| *Unsuccessful* | 30.53 | 5.22 | 30.69 | 6.64 | 32.75 | 5.75 | 32.17 | 7.71 |
|  | **Visit 1 (N = 25)** | | **Visit 2 (N = 23)** | | **Visit 3 (N = 22)** | | **Visit 4 (N = 22)** | |
| ***^AB^LoC – Chance*** | *Mean* | *SD* | *Mean* | *SD* | *Mean* | *SD* | *Mean* | *SD* |
| *Successful* | 17.70 | 7.24 | 15.60 | 7.29 | 16.90 | 8.27 | 17.60 | 9.37 |
| *Unsuccessful* | 17.33 | 6.83 | 15.69 | 5.68 | 16.58 | 5.14 | 16.92 | 5.82 |
|  | **Visit 1 (N = 25)** | | **Visit 2 (N = 23)** | | **Visit 3 (N = 22)** | | **Visit 4 (N = 22)** | |
| ***^AB^LoC – Others*** | *Mean* | *SD* | *Mean* | *SD* | *Mean* | *SD* | *Mean* | *SD* |
| *Successful* | 16.80 | 7.10 | 14.20 | 7.01 | 16.50 | 7.63 | 15.70 | 6.99 |
| *Unsuccessful* | 17.20 | 7.03 | 16.46 | 4.89 | 15.83 | 6.10 | 15.42 | 5.76 |
|  | **Visit 1 (N = 10)** | | **Visit 2 (N = 8)** | | **Visit 3 (N = 5)** | | **Visit 4 (N = 3)** | |
| ***^C^LoC – Internality*** | *Mean* | *SD* | *Mean* | *SD* | *Mean* | *SD* | *Mean* | *SD* |
| *Successful* | 14.00 | 6.86 | 15.25 | 5.38 | 13.33 | 8.51 | 17.00 | 7.07 |
| *Unsuccessful* | 16.20 | 4.49 | 11.50 | 4.66 | 7.50 | 2.12 | 6.00 | . |
|  | **Visit 1 (N = 10)** | | **Visit 2 (N = 8)** | | **Visit 3 (N = 5)** | | **Visit 4 (N = 3)** | |
| ***^C^LoC – Chance*** | *Mean* | *SD* | *Mean* | *SD* | *Mean* | *SD* | *Mean* | *SD* |
| *Successful* | 21.60 | 7.09 | 20.75 | 6.85 | 22.00 | 9.54 | 17.50 | 7.78 |
| *Unsuccessful* | 18.80 | 4.71 | 15.25 | 7.85 | 7.00 | 1.41 | 8.00 | . |
|  | **Visit 1 (N = 10)** | | **Visit 2 (N = 8)** | | **Visit 3 (N = 5)** | | **Visit 4 (N = 3)** | |
| ***^C^LoC – Doctors*** | *Mean* | *SD* | *Mean* | *SD* | *Mean* | *SD* | *Mean* | *SD* |
| *Successful* | 8.60 | 5.59 | 9.25 | 2.63 | 10.67 | 3.21 | 8.50 | 2.12 |
| *Unsuccessful* | 6.20 | 5.02 | 8.00 | 5.77 | 10.50 | 6.36 | 3.00 | . |
|  | **Visit 1 (N = 10)** | | **Visit 2 (N = 8)** | | **Visit 3 (N = 5)** | | **Visit 4 (N = 3)** | |
| ***^C^LoC – Others (Non-Doctors)*** | *Mean* | *SD* | *Mean* | *SD* | *Mean* | *SD* | *Mean* | *SD* |
| *Successful* | 15.40 | 7.06 | 16.50 | 4.73 | 18.33 | 5.51 | 17.50 | 4.50 |
| *Unsuccessful* | 12.60 | 9.74 | 14.00 | 10.10 | 17.50 | 12.02 | 6.00 | . |
| SE = Self-efficacy  LoC = Locus of control  ^AB^Able bodied participants  ^C^CNP participants  Note: There is no standard deviation for “unsuccessful” CNP participants for visit 4 because the sample size was 1 | | | | | | | | |
